# Metabolic engineering of *Bacillus subtilis* toward the efficient and stable production of C_30_-carotenoids

**DOI:** 10.1186/s13568-023-01542-x

**Published:** 2023-04-29

**Authors:** Oriana Filluelo, Jordi Ferrando, Pere Picart

**Affiliations:** Faculty of Pharmacy and Food Science Technology, Department of Biology, Healthcare and the Environment, Microbiology Section, University of Barcelona, Avinguda Joan XXIII, 27-31, Barcelona, 08028 Spain

**Keywords:** *B. subtilis*, C_30_ carotenoids, CRISPR-Cas9, Metabolic engineering

## Abstract

Commercial carotenoid production is dominated by chemical synthesis and plant extraction, both of which are unsustainable and can be detrimental to the environment. A promising alternative for the mass production of carotenoids from both an ecological and commercial perspective is microbial synthesis. To date, C_30_ carotenoid production in *Bacillus subtilis* has been achieved using plasmid systems for the overexpression of biosynthetic enzymes. In the present study, we employed a clustered regularly interspaced short palindromic repeat-Cas9 (CRISPR-Cas9) system to develop an efficient, safe, and stable C_30_ carotenoid-producing *B. subtilis* strain, devoid of plasmids and antibiotic selection markers. To this end, the expression levels of *crtM* (dehydrosqualene synthase) and *crtN* (dehydrosqualene desaturase) genes from *Staphylococcus aureus* were upregulated by the insertion of three gene copies into the chromosome of *B. subtilis*. Subsequently, the supply of the C_30_ carotenoid precursor farnesyl diphosphate (FPP), which is the substrate for CrtMN enzymes, was enhanced by expressing chromosomally integrated *Bacillus megaterium*-derived farnesyl diphosphate synthase (FPPS), a key enzyme in the FPP pathway, and abolishing the expression of farnesyl diphosphate phosphatase (YisP), an enzyme responsible for the undesired conversion of FPP to farnesol. The consecutive combination of these features resulted in a stepwise increased production of C_30_ carotenoids. For the first time, a *B. subtilis* strain that can endogenously produce C_30_ carotenoids has been constructed, which we anticipate will serve as a chassis for further metabolic engineering and fermentation optimization aimed at developing a commercial scale bioproduction process.

**Supplementary Information:**

The online version contains supplementary material available at 10.1186/s13568-023-01542-x.

## Key points

• Overexpression of chromosomally integrated *crtMN* genes improved C_30_ carotenoid production
• Overexpression of FPPS and branch pathway attenuation further enhanced C_30_ carotenoid yield
• A stable plasmid-less, marker-less C_30_ carotenoid-producing *B. subtilis* strain was constructed

## Introduction

Terpenoids (also known as isoprenoids) constitute one of the largest and structurally most diverse groups of natural products with diverse biological functions (Zhang and Hong 2020). An economically important class of terpenoids are the carotenoids, which are ubiquitous lipid-soluble pigments responsible for the red, yellow, and orange colors of plants, algae, fungi, and bacteria (Cardoso et al. 2017). Although commercial carotenoid production is dominated by chemical synthesis and plant extraction, these processes are not sustainable or ecological. Carotenoids are chemically synthesized under harsh conditions, generating byproducts and hazardous waste, whereas sourcing carotenoids from plant extracts is generally dependent on the seasons and geographic areas, which cannot always be standardized (Siziya et al. 2022). Therefore, microbial production is emerging as one of the most promising safe and environmentally friendly options to satisfy the fast-growing demands for carotenoids (Siziya et al. 2022).

*B. subtilis* is generally recognized as safe (GRAS), has a high growth rate, and is easy to genetically manipulate and cultivate, with a wide substrate range (Earl, 2008; Schallmey et al., 2004). In addition, it is one of the highest producer of isoprene (the smallest terpenoid) among eubacteria, thus constituting an ideal microbial host for use as a terpenoid cell factory (Kuzma et al. 1995; Wagner et al. 2000; Julsing et al. 2007; Moser and Pichler 2019; Guan et al. 2015). This bacterium is able to initiate terpenoid biosynthesis from simple carbon sources through the methylerythritol 4-phosphate (MEP) pathway, a route with eight enzymatic reactions leading to the synthesis of isopentenyl diphosphate (IPP; C5) and dimethylallyl diphosphate (DMAPP; C5), the universal precursors of all terpenoids (Guan et al. 2015). The consecutive condensation of IPP and DMAPP is catalyzed by prenyl diphosphate synthase (*IspA*) to produce starting precursors for the synthesis of different classes of terpenoids: geranyl diphosphate (GPP; C10), a monoterpenoid precursor; farnesyl diphosphate (FPP; C15) for the production of sesquiterpenoids, triterpenoids and C_30_-carotenoids, and geranylgeranyl diphosphate (GGPP; C20), the precursor of diterpenoids and carotenoids (Moser and Pichler 2019). Most carotenoids contain a 40-carbon backbone (C_40_ carotenoids), including β-carotene, lycopene and astaxanthin, whereas those with 30-carbon backbones (C_30_ carotenoids), such as 4,4’-diaponeurosporene (DNP) and 4,4’- diapolycopene (DLP), are synthesized by a limited group of bacteria, including *Staphylococcus aureus* (Marshall and Wilmoth 1981), and *Heliobacteria spp*. (Takaichi et al. 1997). Genes responsible for C_30_ carotenoid biosynthesis in *S. aureus* have been characterized (Pelz et al. 2005; Wieland et al. 1994).) The first dedicated enzyme in the C_30_ carotenoid synthetic pathway is CrtM (dehydrosqualene synthase), which catalyzes the head-to-head condensation of two molecules of FPP to dehydrosqualene. The enzyme CrtN (dehydrosqualene desaturase) then converts dehydrosqualene to the yellow C_30_ carotenoid, DNP, a relatively unstable compound that can suffer further oxidation by CrtMN to yield DLP. The action of these two enzymes probably constitutes the most common route of C_30_ carotenoid biosynthesis in bacteria. Notably, these yellow pigments have attracted interest from the pharmaceutical industry owing to their powerful antioxidant activities (Yoshida et al. 2009), as well as their role as immunomodulators, significantly enhancing the immune system (Jing et al. 2017, 2019; Liu et al. 2016, 2017). Consequently, microbial cell engineering approaches aimed at improving C_30_ carotenoid yields are required to achieve industrial-scale production.

**Fig. 1:**
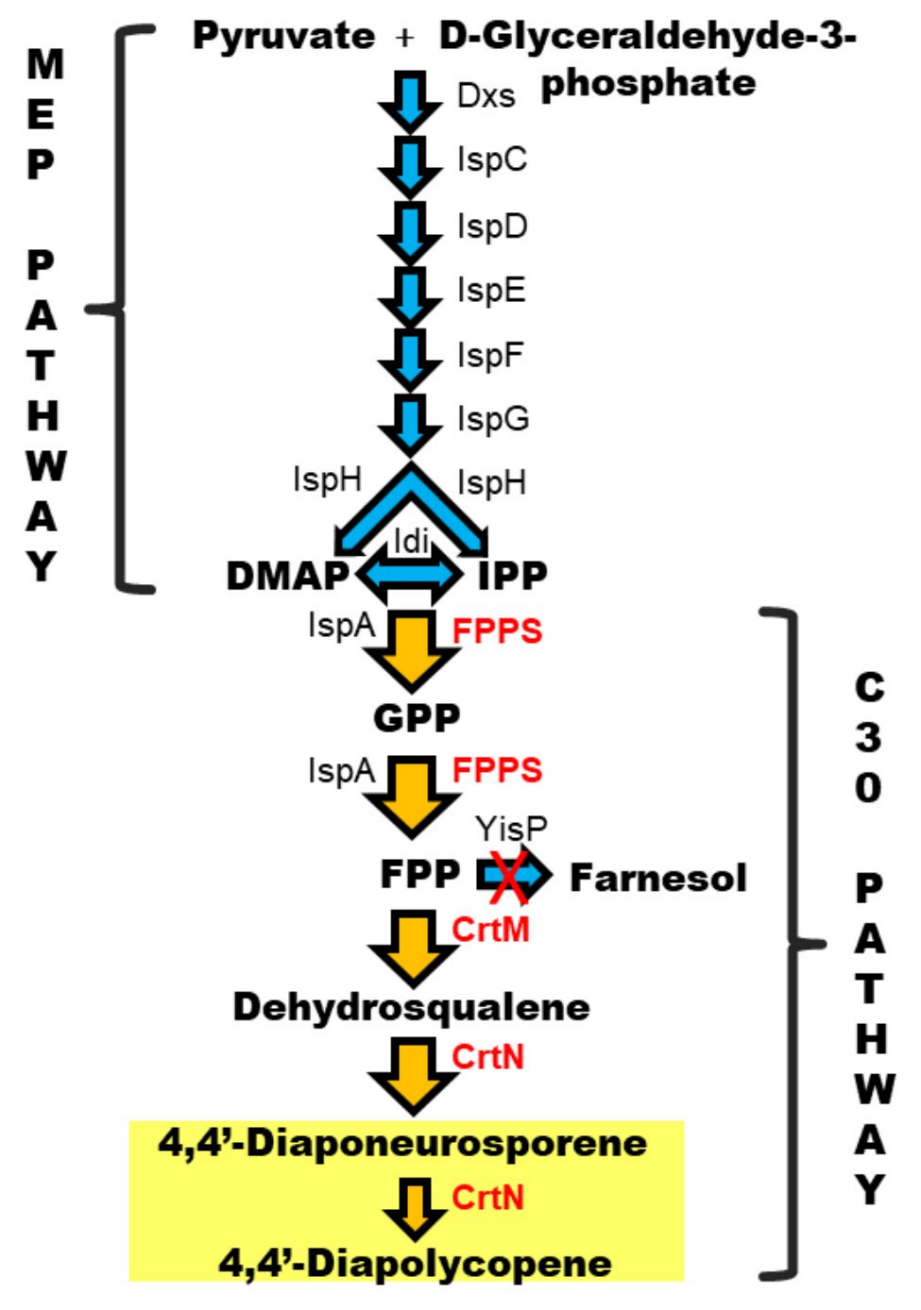
Metabolic pathways associated with terpenoid biosynthesis in *B. subtilis* and engineering strategies for the production of yellow C_30_ carotenoids 4,4’-diaponeurosporene and 4,4’-diapolycopene (C_30_ pathway). Foreign genes are marked in red. Yellow arrows outlined in black indicate the reactions reinforced by chromosomic overexpression of the *fpps* gene (farnesyl diphosphate synthase) from *B. megaterium* DSM 319, *crtM* (squalene desaturase) and *crtN* (dehydrosqualene desaturase or diapophytoene desaturase) genes from *S. aureus*, and deletion of the *yisP* (farnesyl diphosphate phosphatase) gene, yielding the C_30_ carotenoid pigments 4,4’-diaponeurosporene and 4,4’-diapolycopene. Enzymes in the MEP (Methylerythritol 4-phosphate) pathway: 1-deoxy-D-xylulose-5-phosphate synthase (*Dxs*); 1-deoxy-D-xylulose-5-phosphate reductoisomerase or 2-C-methyl-D-erythritol 4-phosphate synthase (*Dxr*, also known as *IspC*); 2-C-methyl-D-erythritol 4-phosphate cytidylyltransferase (*IspD*); 4-(cytidine 5′-diphospho)-2-C-methyl-D-erythritol kinase (*IspE*); 2-C-methyl-D-erythritol 2,4-cyclodiphosphate synthase (*IspF*); (E)-4- hydroxy-3-methylbut-2-enyl-diphosphate synthase (*IspG*); 4-hydroxy-3- methylbut-2-enyl diphosphate reductase (*IspH*); and isopentenyl-diphosphate delta-isomerase (*Idi*). Geranyltransferase (*IspA*, also known as *YqiD*) refers to the *B. subtilis* gene responsible for the supply of GPP (geranyl diphosphate) and FPP (farnesyl diphosphate) precursors

To date, the metabolic engineering of *B. subtilis* toward enhanced C_30_ carotenoid production has focused on using two-plasmid systems comprising pHY_crtMN (Yoshida et al. 2009), mediating *crtMN* gene overexpression under tetracycline selection, and xylose-inducible pHCMC04G (Xue et al. 2015), mediating stable overexpression of all MEP pathway enzymes under non-selection conditions (Abdallah et al. 2020). However, two-plasmid systems may impose a metabolic burden on the host cells, leading to lower growth rates and increased productivity costs (Wu et al. 2016). Another drawback is the high-cost of the inducer compounds and, more importantly, the requirement for antibiotic usage, which is restricted by governmental regulations and can thus hinder the establishment of a commercially viable industry. On the other hand, very little work has been done to explore the effects of modulating *crtMN* gene expression and other competing branch pathways (which can limit FPP availability) on C_30_ carotenoid production, leaving room for improvement. In this study, we initially compared the expression levels of plasmid-based and chromosomally integrated *crtMN* genes, and then implemented CRISPR-Cas9-based metabolic engineering strategies to achieve an efficient C_30_ carotenoid-producing strain of *B. subtilis*, a bacterium that naturally produces yellow pigments (Fig. 1). Thus, with the aim of increasing the supply of the carotenoid precursor FPP, we planned (i) to introduce a chromosomally integrated copy of FPPS (farnesyl diphosphate synthase), and (ii) to abolish the activity of a competing branch pathway that uses FPP. With this approach, it was envisaged that we could construct a stable and efficient C_30_ carotenoid-producing *B. subtilis* strain that was plasmid- and marker-free, an attribute of paramount importance for its potential development into a commercially viable bioprocess.

## Materials and methods

### Bacterial strains

The *E. coli* NEB® turbo strain (New England Biolabs) was used as the host strain for routine molecular cloning and plasmid construction operations, and *B. subtilis* KO7-S (Bacillus Genetic Stock Center), an asporogenous strain with seven inactivated protease genes, was used as a host strain for C_30_ carotenoid production. DNA isolation and.

manipulations were carried out using standard protocols. The bacterial strains employed in this research are listed in Table 1.

**Table 1:** Bacterial strains and plasmids used in this study

Strain	Genotype or description	Source/Reference
*E. coli NEB®* turbo	*F’ proA + B + lacIq ∆lacZM15 / fhuA2 ∆(lac-proAB) glnV galK16 galE15 R(zgb-210::Tn10)*TetS	Laboratory stock
*B. megaterium DSM 319*	Source of *fpps* gene	DSM
*B. subtilis* 168 KO7-S	*ΔnprE ΔaprE Δepr Δmpr ΔnprB Δvpr Δbpr ΔsigF*	BGSC
BsMN0	*B. subtilis* KO7-S strain harboring the plasmid pHY_crtMN	This study
BsMN1	Contains one *crtMN* gene-copy integrated into the genome	Ferrando et al., 2023
BsMN2	Contains two *crtMN* gene-copies integrated into the genome	Ferrando et al., 2023
BsMN3	Contains three *crtMN* gene-copies integrated into the genome	Ferrando et al., 2023
BsMN4	*B. subtilis* KO7-S strain harboring the plasmid pBS0E_crtMN	This study
BsMN5	BsMN3 strain with an *fpps* gene from *B. megaterium* replacing the *sigX* gene	This study
BsMN6	BsMN5 strain with a truncated copy of the *yisP* gene	This study
**Plasmid**	**Description**	**Source/Reference**
pHY_crtMN	Plasmid pHY300PLK containing the *crtMN* operon from *Staphylococcus aureus*	Yoshida et al. 2009
pBS0E	Plasmid containing the xylose-inducing promoter xylose-repressor system	Popp et al. 2017
pJOE8999	PmanP-cas9, pUC, pE194ts, *kanr*	Altenbuchner 2016
pJOE8999_VG_MN	Plasmid used to replace the *spoVG* locus for the *crtMN genes*	Ferrando et al., 2023
pBS0E_crtMN	Plasmid containing *crtMN* genes under the control of xylose-inducing promoter	This study
pJOE8999.sg_sigX_fpps	Plasmid used to replace the *sigX* locus for the *fpps* gene	This study
pJOE8999.sg_ΔyisP	Plasmid used to delete the *yisP* gene	This study

### Medium and culture conditions

*E. coli* strains were cultured in Luria-Bertani (LB) medium at 37 ºC, while *B. subtilis* KO7-S strains were grown in Tryptic Soy Broth (TSB) (17 g/l tryptone, 3 g/l soytone, 2.5 g/l dextrose, 5.0 g/l NaCl, 2.5 g/l K_2_HPO_4_) or *Bacillus subtilis* 1 (BS1) medium, typically used in industrial fermentation (Wenzel et al. 2011). The BS1 medium contained standard salts (in g/l: 2 (NH4)2SO4; 18.3 K2HPO4·3H2O; 6 KH2PO4; 1 Na+-citrate; 0.2 MgSO4·7H2O), trace metals (in mg/l: 120 FeSO4·7H2O; 30 MnSO4·H2O; 12 CuSO4·5H2O; 12 ZnCl2) and was supplemented with 12 g sucrose/l and 18 g soybean meal/l (Sigma Aldrich). All strains were incubated at 37 ºC on a rotatory shaker at 200 rpm. When necessary, the growth media were supplemented with antibiotics at the following concentrations: 30 µg/ml kanamycin for *E. coli*, and 6 µg/ml kanamycin or 10 µg/ml tetracycline or 2 µg/ml erythromycin for *B. subtilis*. To induce the CRISPR-Cas9 system in *B. subtilis* cells, 0.5% D-mannose was added.

### Plasmid construction and primers

The plasmids used in this study are listed in Table 1 and the primers in Table S1. For the insertion of *crtMN* genes into the pBS0E vector (Popp et al. 2017), the pHY_crtMN plasmid (Yoshida et al. 2009) was used as a template to amplify *crtMN* genes using primers P1F/P1R. The resulting DNA amplicon was treated with *Eco*RI and *Spe*I and cloned into the replicative plasmid pBS0E for the construction of the xylose-inducible pBS0E_crtMN vector. CRISPR-Cas9-mediated genome editing in *B. subtilis* was performed using the pJOE8999 vector as the parental plasmid, according to a previously described method (Altenbuchner 2016).

### **Chromosomal integration of the *fpps* gene**

To generate the *sigX* gene replacement by the *fpps* gene, oligonucleotides for 20 pb gRNA (TS1F and TS1R) were synthesized and ligated to *Bsa*I-digested pJOE8999. *sigX*-targeting gRNA containing pJOE8999 was named pJOE8999.g_sigX. A repair template for *fpps* integration into the *sigX* gene was constructed in vitro by overlap extension PCR of three fragments as follows: the 800-bp upstream flanking genomic region of *sigX* (P2F/P2R primers) followed by the *fpps* gene (P3F/P3R primers) and the 800-bp downstream flanking genomic region of *sigX* (P4F/P4R primers). Homologous arms were amplified using the *B. subtilis* KO7-S chromosome as a template, while the *fpps* gene was amplified using genomic DNA from *B. megaterium* DSM 319. The fused fragment was digested with *Sfi*I and then ligated into pJOE8999.g_sigX, which had also been digested with *Sfi*I to obtain the editing plasmid pJOE8999.g_sigX_fpps, used for FPPS overexpression.

### **Deletion of the *yisP* gene**

To generate the *yisP* knockout mutant, a procedure similar to the one described above was performed. Primers TS2F and TS2R targeting the *yisP* gene were synthesized and ligated to the vector, thus obtaining plasmid pJOE8999.g_yisP. A 1.6 kb repair template, containing the 800-bp upstream region and 800-bp downstream region of the *yisP* gene, was PCR-amplified using the *B. subtilis* KO7-S genome as a template. Primer sets P5F/P5R and P6F/P6R were used to amplify each fragment and fused together by overlapping PCR. The repair template was further digested with *Sfi*I for ligation with pJOE8999.g_yisP to obtain the editing plasmid pJOE8999.sg_ΔyisP, which was used to delete the *yisP* gene.

### Transformation and plasmid curing

The well-established plasmids (1 µg) were then transformed to *B. subtilis* KO7-S according to the standard methods described by Yasbin and coworkers (Yasbin et al. 1975). For the CRISPR-Cas9-induced genome editing, the resulting transformants were passaged three times on LB agar plates (without any antibiotics) at 50 °C for 24 h to cure the plasmid. The colonies were confirmed as cured of the editing plasmid by streaking them onto LB agar plates containing kanamycin or no antibiotics; plasmid cured colonies fail to grow at 37 °C. To confirm whether the desired insertion or deletion in the genome of *B. subtilis* had been performed, a colony PCR was conducted to amplify the target fragments from the bacterial chromosome and validated by further Sanger sequencing.

### **Extraction of carotenoids from *B. subtilis***

Carotenoids were extracted from the engineered *B. subtilis* cells according to the literature (Xue et al. 2015) with some modifications. Briefly, recombinant strains were inoculated in 50 ml TSB at an optical density (OD_600_) of 0.05 and cultured for 24 h at 37 ºC (250 rpm). In the case of xylose-inducing experiments, 1% xylose was added at an OD_600_ of 0.6, and strains were then cultured for an additional 24 h in the same conditions. Samples were collected by centrifugation at 8000* g* for 15 min and washed with 1 ml TE buffer (10 mM Tris/HCl, 1 mM EDTA, pH 8.0). The cells were resuspended in 500 µl TE buffer. To extract the carotenoids, cell suspensions were lysed with 25 µl of 100 mg/ml lysozyme, followed by incubation for 15 min at 37 °C. The cell lysate was then transferred into a glass tube, covered in aluminum foil to avoid light exposure, and centrifuged for 20 min at 2100 g. The supernatant was removed, and 1 ml acetone was added to the pellets. These were vortexed for 4 min, heated for 2 min in a water-bath at 55ºC, and then vortexed again for 2 min. After centrifugation at 2300 g for 15 min, the supernatants were collected and transferred to a new glass tube. The acetone extraction was repeated four times. Next, the acetone extracts were evaporated, and the remaining carotenoids were dissolved in 100 µl acetone and collected in HPLC vials, prior to their analysis using an HPLC system. Cell dry weight was determined by pelleting and drying a fraction of the culture.

### HPLC analysis of carotenoids

Carotenoid extracts were analyzed with a Shimadzu HPLC system equipped with a Gemini® NX-C18 column (5 μm, 110 Å, 250 × 4.60 mm) and a UV/VIS detector at 25 °C. The mobile phase consisted of acetonitrile and water (85:15%) at a flow rate of 2 ml/min. DNP and DLP were identified from their absorption spectra and quantified by comparing their peak areas using an standard calibration curve prepared with known amounts of β-carotene (quantified by absorbance), then multiplying by the molar extinction coefficient (ε) of β-carotene (138,900 M − 1 cm − 1 at 450 nm) (Britton et al., 2004), and dividing by the ε value for the carotenoid in question (147,000 M − 1 cm − 1 at 440 nm for DNP, 185,000 M − 1 cm − 1 at 470 nm for DLP) (Furubayashi et al. 2014). Production weights of carotenoids were then normalized to the dry cell weight (DCW) of each culture.

## Results

### **Dependence on the*crtMN*gene copy number in C**_**30**_**carotenoid production**

A set of plasmid-less, marker-free *B. subtilis* strains harboring one (BsMN1), two (BsMN2) or three copies (BsMN3) of *crtMN* genes in their chromosomes under the control of the constitutive *spoVG* promoter were previously constructed by our research group, but not characterized (Ferrando et al. 2023). Therefore, to investigate the effect of multiple *crtMN* gene copy expression on the intracellular accumulation of C_30_ carotenoids, cells of a stationary overnight culture in TSB were diluted to an OD_600_ of 0.05 in TSB and grown in shake flasks at 220 rpm and 37 ºC for 24 h. Then, samples were taken to quantify both the DCW and the total amounts of DNP and DLP by HPLC. The latter were calculated as mg/g DCW to allow comparison between the strains. The parental *B. subtilis* strain (BsMN0) containing only the pHY_crtMN plasmid was used as a control.

**Fig. 2:**
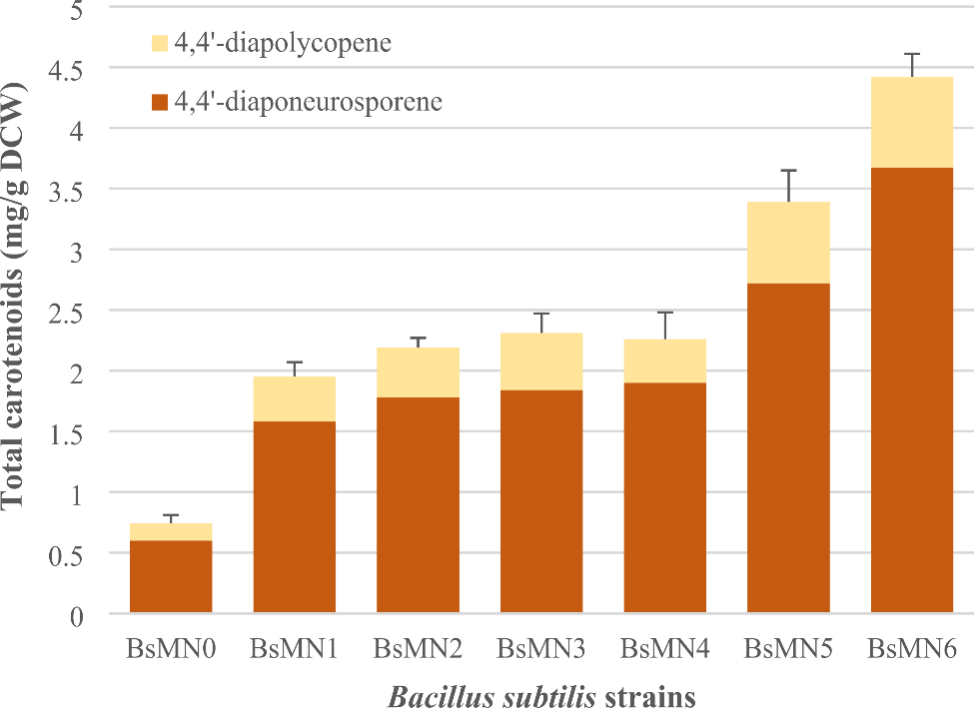
Quantitative analysis of C_30_ carotenoids produced by engineered *B. subtilis* strains. Samples were analyzed by HPLC after C_30_ carotenoid extraction with acetone. Quantification of each C_30_ carotenoid was performed comparing peak areas with the standard reference curve, and then normalized to the dry cell mass of each culture. The amount of DNP is indicated in orange and the amount of DLP in yellow. The experiments were performed in triplicate

After 24 h of growth, all engineered *B. subtilis* strains had an OD_600_ of 7–8, with DCW values of 1.23–1.47 g/L, showing a slight increase in DCW as the *crtMN* gene copy number increased (Table 2). HPLC chromatogram analysis revealed two major peaks at 450 nm, which eluted at 2.4 and 2.8 min, with absorption spectra for each peak identical to those of DLP and DNP, respectively (Fig. S1) (Takaichi 2000; Takaichi et al. 1997). As the two peaks were present in the chromatograms of all samples, both compounds were calculated individually as well as together as total carotenoids, with the results provided in Fig. 2; Table 2. Surprisingly, the BsMN1 strain harboring a single copy of *crtMN* genes produced a titer of 2.40 ± 0.13 mg/L carotenoids with a yield of 1.95 ± 0.12 mg/g DCW, which was already more than a 2-fold increase in total carotenoid production compared to strain BsMN0 containing the pHY_crtMN plasmid (0.74 ± 0.12 mg/g DCW). We observed that DCW and carotenoid yield slightly increased with increasing *crtMN* copy number and the highest titer of 3.30 ± 0.11 mg/L carotenoids was achieved in BsMN3, with a yield of 2.31 ± 0.16 mg/g DCW, which constituted a 3.12-fold increase in carotenoid production compared to BsMN0 (Fig. 2 and Table 2). The yield obtained in BsMN0 was comparable with previously reported values (Xue et al., 2015; Abdallah et al. 2020), which demonstrates the feasibility and robustness of the comparative studies.

**Table 2:** Comparison of dry cell weight, titer and yield of C_30_ carotenoids produced by engineered *B. subtilis* strains and relative increase compared to the control strain

Strain	DCW (g/L culture)	Titer Carotenoids (mg/L culture)	Yield Carotenoids (mg/g DCW)^a^	Relative increase^b^
BsMN0	1.36 ± 0.09	1.01 ± 0.08	0.74 ± 0.07	1
BsMN1	1.23 ± 0.13	2.40 ± 0.13	1.95 ± 0.12	2.64
BsMN2	1.35 ± 0.05	2.96 ± 0.07	2.19 ± 0.08	2.96
BsMN3	1.43 ± 0.06	3.30 ± 0.11	2.31 ± 0.16	3.12
BsMN4	1.87 ± 0.16	4.22 ± 0.23	2.26 ± 0.32	3.05
BsMN5	1.32 ± 0.09	4.49 ± 0.19	3.39 ± 0.33	4.58
BsMN6	1.47 ± 0.08	6.51 ± 0.12	4.42 ± 0.19	5.97
BsMN6^c^	2.98 ± 0.14	9.11 ± 0.36	3.20 ± 0.24	NA^d^

^a^ The total amount of carotenoids was measured in triplicate (± standard deviation)

^b^ The relative increase is calculated as the amount of carotenoids produced in the engineered *B. subtilis* strain divided by the amount of carotenoids produced in the control strain (BsMNO) harboring the pHYCrtMN vector

^c^ Strain cultured in BS1 medium

^d^ Not applicable

The low carotenoid yield obtained in BsMN0 suggested that *crtMN* genes are poorly expressed through the pHY_crtMN plasmid. To test this hypothesis, we cloned the *crtMN* genes in the xylose-inducible medium copy number pBS0E plasmid (Popp et al. 2017), which is particularly useful for overcoming bottlenecks in protein overproduction generated by limited expression of targeted genes (Toymentseva et al. 2012). The *B. subtilis* strain bearing the pBS0E_crtMN plasmid (BsMN4) showed a higher cell growth compared to BsMN0 – BsMN3 strains, with a DCW of 1.87 g/L, probably due to the addition of an extra carbon source (D-xylose inducer) to the media. As expected, BsMN4 exhibited a notable increase in carotenoid yield (3.05-fold) compared to BsMN0, demonstrating a higher expression of *crtMN* genes through this plasmid (Table 2). More importantly, the yield obtained for strain BsMN4 was similar to that of BsMN3, indicating that plasmid-bearing and multicopy strains had a comparable performance.

### Optimization of the C_30_ carotenoid biosynthetic pathway

**Fig. 3:**
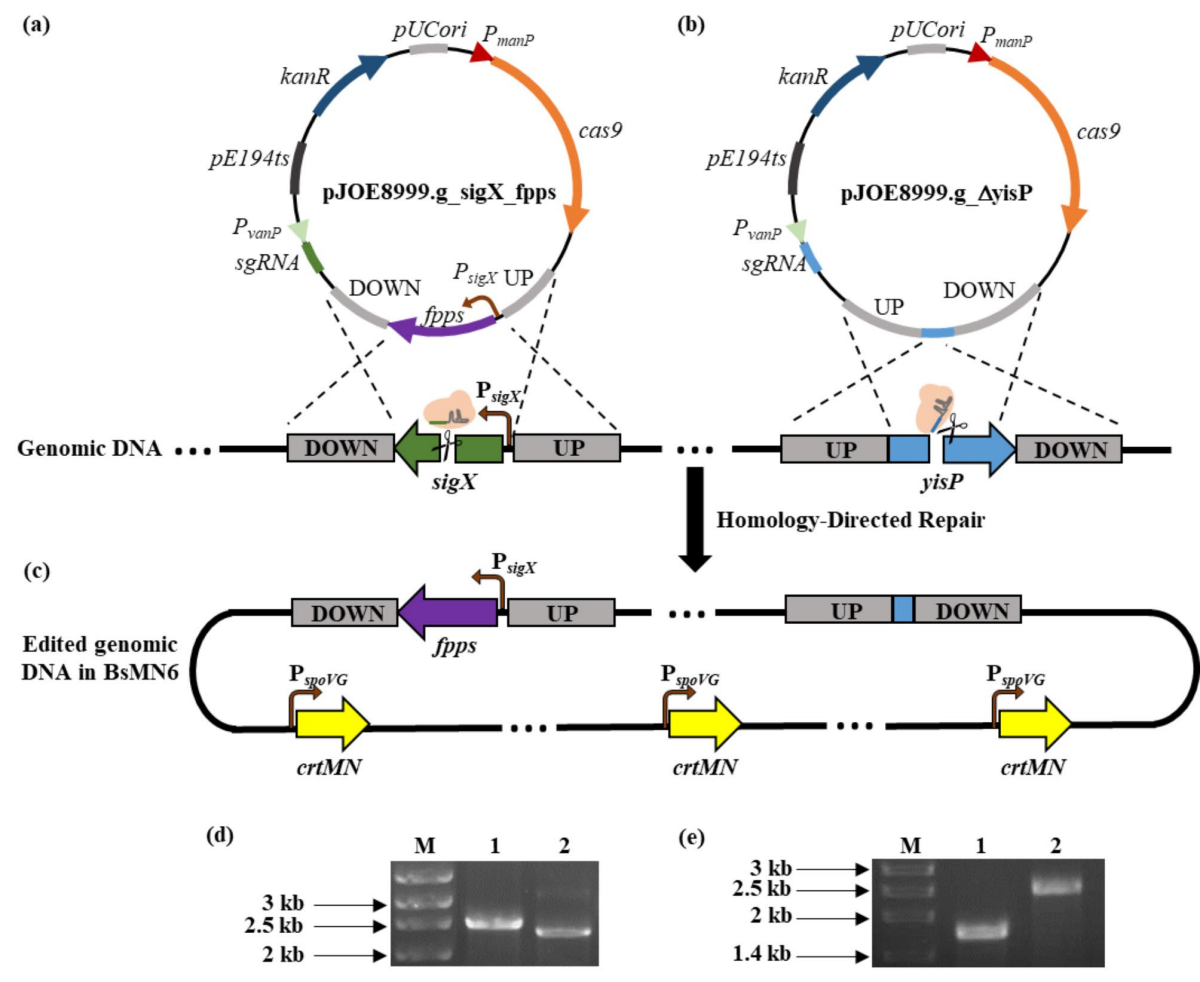
Engineering of the genome-integrated farnesyl diphosphate synthase (FPPS) and disruption of farnesyl diphosphate phosphatase (YisP) in *B. subtilis*. (**a**) pJOE8999.g_sigX_fpps was designed to allow the replacement of the *sigX* gene from *B. subtilis* by the *fpps* gene from *B. megaterium* under the control of a strong promoter P_*sigX*_. (**b**) pJOE8999.g_ΔyisP was constructed for the disruption of the *yisP* gene from *B. subtilis* (**c**) Upon transformation, the resulting *B. subtilis* strain harboring both genomic modifications along with three gene-copies of the *crtMN* genes under the control of the constitutive promoter P_*sigX*_ was designated as BsMN6. (**d**) Confirmation of the *sigX* gene replacement by *fpps* in the BsMN5 strain. Lane 1 corresponds to an amplification band of 2.5 kb using primers P2F/P4R to verify *fpps* integration at the *sigX* locus site in BsMN5. Lane 2 corresponds to an amplification band of 2.25 kb using the same primers in recipient strain BsMN3. M corresponds to the molecular marker weight. (**e**) Confirmation of the *yisP* gene disruption in strain BsMN6. Lane 1 corresponds to an amplification band of 1.75 kb using primers P5F/P6R to verify *yisP* deletion in BsMN6. Lane 2 corresponds to an amplification band of 2.5 kb using the same primers in recipient strain BsMN5. M corresponds to the molecular marker weight

In the C_30_ carotenoid metabolic pathway in *B. subtilis*, farnesyl diphosphate synthase (IspA) converts the universal terpenoid precursors DMAPP and IPP to FPP, which is the substrate for CrtMN enzymes in C_30_ carotenoid biosynthesis (Fig. 1). In order to further improve the production of C_30_ carotenoids, we aimed to increase the FPP supply, as studies report that enhanced FPP availability drives metabolic flux toward their synthesis (Xue et al. 2015; Abdallah et al. 2020; Song et al. 2021). This has been achieved previously by introducing either an extra copy of *ispA* to release the theoretical bottleneck within the metabolic pathway or an improved variant of the enzyme with enhanced catalytic properties (Zhao et al. 2013). In the present study, farnesyl diphosphate synthase (encoded by the *fpps* gene) from *B. megaterium* DSM 319, which is an active highly specific enzyme exclusively yielding FPP (Hartz et al., 2018), was overexpressed to enhance the FPP pool. To this end, plasmid pJOE8999.sigX_fpps was constructed for the replacement of the *sigX* gene of BsMN3 (codifying for sigma factor SigX) with the *fpps* gene, setting the expression of the encoded FPPS under the control of a strong *sigX* promoter (Song et al. 2016), and strain BsMN5 was generated (Fig. 3a). The insertion of the *fpps* gene in cured transformant cells was confirmed by diagnostic PCR (Fig. 3d) and further Sanger sequencing. Fermentation studies revealed a remarkable 46.8% increase in the production of C_30_ carotenoids compared with BsMN3 (Fig. 2and Table 2). Additionally, BsMN5 grew at a similar rate to the parental strain BsMN3, indicating that the overexpression of FPPS did not affect cell growth in TSB medium. Based on these results, we surmised that heterologous expression of FPPS in *B. subtilis* is beneficial for the construction of a high-yielding C_30_ carotenoid-producing strain.

### Branch pathway engineering to increase C_30_ carotenoid production

To provide enough FPP for C_30_ carotenoid biosynthesis, it is crucial to attenuate branch pathways that use this precursor as the starting material. In the biosynthesis of farnesol lipids, each FPP molecule is converted to farnesol by the action of farnesyl diphosphate phosphatase (YisP) (Fig. 1); therefore, this branch pathway was selected as a candidate for engineering. Plasmid pJOE8999_ΔyisP was constructed to knock out a 770-bp fragment of *yisP* in strain BsMN5 and inactivate the function of YisP, thus blocking the synthesis of farnesol in the newly generated strain BsMN6 (Fig. 3b and c). Disruption of the *yisP* gene in resulting transformants was confirmed by PCR amplification, as previously (Fig. 3e), and further verified by sequencing. The positive clone was cured from the plasmid and subjected to fermentation for 24 h to measure the production of DLP and DNP. Again, BsMN6 growth was similar to the parental strain BsMN5, indicating that *yisP* disruption in BsMN6 did not affect cell growth. However, C_30_ carotenoid production in strain BsMN6 was significantly enhanced, being 130.4% relative to BsMN5 after fermentation (Fig. 2; Table 2). Overall, combining the simultaneous overexpression of farnesyl diphosphate synthase, dehydrosqualene synthase, and dehydrosqualene desaturase encoded by *fpps*, *crtM* and *crtN*, respectively, and the disruption of the *yisP* gene positively affected C_30_ carotenoid production in strain BsMN6, which was up to 6-fold higher compared to the control strain BsMN0 (Fig. 2; Table 2).

### Stability of BsMN6 in C_30_ carotenoid production and its cultivation in industrial fermentation medium

**Fig. 4:**
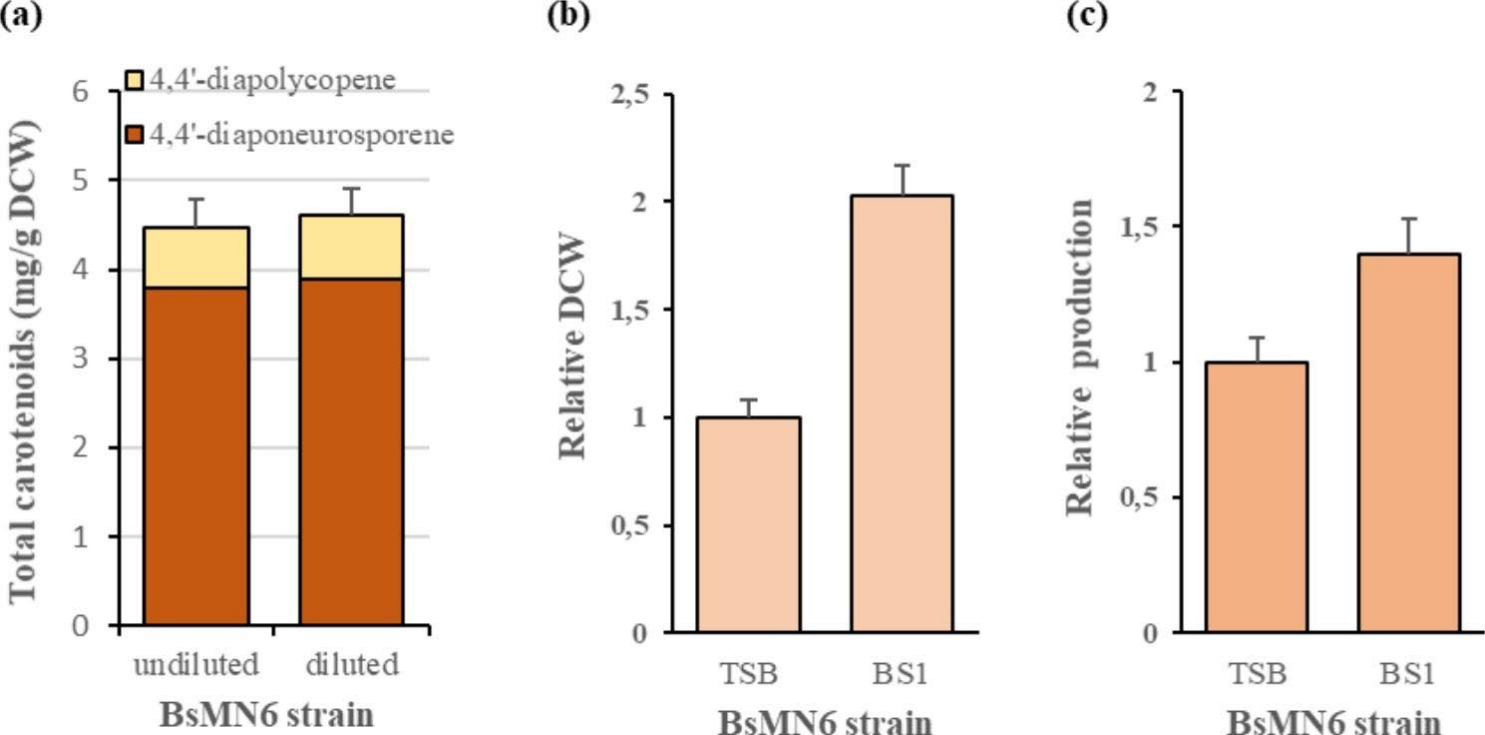
Stability and C_30_carotenoid production in strain BsMN6. (**a**) C_30_ carotenoid production in the BsMN6 strain diluted 1000-fold and grown to the stationary phase, repeated 5 times, without antibiotics in TSB media. (**b**) Relative DCW and (**c**) relative titers of C_30_ carotenoids produced by strain BsMN6 cultured in TB and BS1 media, after 24 h of fermentation. The error bars represent the average ± standard deviation of three biological replicates

The stability of C_30_ carotenoid production in strain BsMN6 without antibiotic selection was tested. An overnight culture of BsMN6 in TSB was diluted 1:1000 in the same medium. The cells were grown in shake flasks at 37 °C to the stationary phase and diluted again 1000-fold. This was repeated five times and in the last transfer, when the stationary phase was reached, the strain was cultured again in TSB and the C_30_ carotenoid yield was determined. As shown in Fig. 4a, BsMN6 produced similar levels of C_30_ carotenoids for at least 50 generations (every round of growth to stationary phase corresponds to about ten generations without antibiotic supplementation, calculated by dividing the length of the exponential growth phase (about 300 min) by the doubling time of BsMN6 (approximately 30 min) in TSB medium), demonstrating that BsMN6 achieved a high yield of C_30_ carotenoids with stable productivity.

To date, recombinant production of C_30_ carotenoids in *B. subtilis* has been exclusively tested by culturing engineered strains in TSB medium at the shake flask level (Yoshida et al. 2009; Xue et al. 2015; Abdallah et al. 2020). However, TSB is a nutritious medium designed to support the growth of a wide variety of microorganisms, and inappropriate for *B. subtilis* fermentation on an industrial scale due to its high cost. We therefore decided to investigate the capacity of strain BsMN6 to accumulate C_30_ carotenoids in BS1, a commonly used industrial bacterial feed (Wenzel et al. 2011). To this end, BsMN6 was cultured for 24 h in TSB and BS1 media before analyzing DCW and C_30_ carotenoid production. As shown in Fig. 4b and c; Table 2, BsMN6 was able to double the cell biomass concentration when grown in BS1 medium (2.98 ± 0.14 g/L culture) compared to the same strain growing in TSB medium (1.47 ± 0.08 g/L culture). Although the yield of C_30_ carotenoids obtained in TSB (4.42 ± 0.19 mg/g DCW) was higher compared to BS1 medium (3.20 ± 0.24 mg/g DCW), the titer of C_30_ carotenoids obtained in the latter was 40% higher than the titer obtained in TSB, reaching a value of 9.11 ± 0.36 mg/L C_30_ carotenoids. This indicates that BS1 medium can stimulate cell growth and had a significantly positive effect on the C_30_ carotenoid titer in comparison with TSB.

## Discussion

The market demand for carotenoids is continuing to grow due to their antioxidant, anti-inflammatory, and anticancer properties. In particular, the biotechnological production of carotenoids to replace artificial pigments is rapidly gaining interest, despite technological, economic, and legislative limitations. *E. coli* and *B. subtilis* strains have been engineered to accumulate C_30_ carotenoids utilizing suitable expression vectors for relevant *crtMN* genes, the overexpression of MEP pathway enzymes, and the concomitant use of antibiotic drugs and plasmids. However, the current trend in industrial bioprocesses is to circumvent the use of antibiotic selection markers by developing marker-free production systems due to concerns derived from the massive overuse of antibiotics. In many areas of biotechnology, restrictions on antibiotic usage have been imposed by regulatory authorities (Mingon et al., 2015). In the present work, we constructed a plasmid-less, marker-free strain of *B. subtilis*, a bacterium that can naturally produce C_30_ carotenoids in the absence of any inducer or antibiotic compound. Optimization steps involving *crtMN* gene dosage and an enhanced supply of the precursor FPP were carried out using the CRISPR-Cas9 system, resulting in the generation of an efficient, safe, and stable C_30_ carotenoid-producing *B. subtilis* strain.

Reliance on the use of plasmids and antibiotic selection markers constitutes a major limiting factor for the implementation of an optimal *B. subtilis* chassis able to execute the functions needed for efficient C_30_ carotenoid production. To bypass this limitation, an interesting option is to maintain the cloned genes by genome integration, thus ensuring high stability in the absence of antibiotic selection pressure. Nevertheless, the main drawback of this approach is that the resulting strains have a low gene dosage unless multiple gene copies are integrated into the genome (Yomantas et al. 2011; Huang et al. 2017; Wang et al. 2004), until reaching expression levels comparable to those of cells carrying multiple copies of a recombinant plasmid. Our study clearly shows that the low copy number pHY_crtMN plasmid (5–15 per cell), a derivative of pHY_300PLK (Ishiwa and Shibahara 1985), is an unfavorable vector for maximizing *crtMN* gene expression. We hypothesize that the reason for the low expression achieved is that *crtMN* genes are the second and third genes transcribed from the promoter of the tetracycline resistant gene (Isamu Maeda personal communication). Within an operon, the expression of a gene at the first position is expected to be higher compared to the gene at the second position, which in turn should be more expressed than a gene at the third position (Lim et al. 2011). In contrast, C_30_ carotenoid production in cells carrying multiple copies of the xylose-inducible medium copy number pBS0E_crtMN plasmid (15–25 per cell) was significantly improved; more importantly, its performance was comparable to the plasmid-less strain harboring three *crtMN* gene copies in the chromosome. Presumably, when these conditions occur, increasing the copy number no longer enhances expression levels (Widner et al. 2000) and the potential bottlenecks in C_30_ carotenoid production rely on the expression of other rate-limiting enzymes in the biosynthetic pathway. Notably, the insertion of three *crtMN* gene copies into the *B. subtilis* chromosome debottlenecked an unexplored rate-limiting step in the C_30_ carotenoid biosynthetic route and at the same time alleviated the need for antibiotic selection for plasmid maintenance. Moreover, its stability and potential ecological safety suggests that the engineered *B. subtilis* strain has great promise as an efficient C_30_ carotenoid cell factory with practical application in industrial settings (García-Moyano et al. 2020; Su et al. 2020).

To further improve the *B. subtilis* carotenoid production capacity, we focused on modulating some of the well-recognized regulatory elements that tightly control the metabolic flux to C_30_ carotenoid biosynthesis from the universal precursors DMAPP and IPP. Specifically, our aim was to enhance the FPP pool and also ameliorate its consumption by removing the competing pathway yielding farnesol. The first attempt to overexpress the *fpps* gene from *B. megaterium* resulted in a significant improvement (1.46-fold) of C_30_ carotenoid production. This result is in accordance with a previous study that achieved 1.36-fold higher carotenoid yields by introducing an extra copy of the homologous *fpps* gene from *B. subtilis* (*ispA*) (Xue et al. 2015). The additional expression of the *fpps* gene from *Saccharomyces cerevisiae* also increased the supply of the precursor FPP (Song et al. 2021). We therefore conclude that the heterologous expression of FPPS from *B. megaterium* increased C30 carotenoid biosynthesis in *B. subtilis*, similarly to the values obtained when an extra copy of the native IspA was overexpressed (Xue et al. 2015). It has also been reported that attenuation of a competing FPP-consuming pathway toward C55 heptaprenyl diphosphate contributed to a 1.15-fold increase in terpenoid synthesis (Song et al. 2021). Accordingly, we assumed that abolishing non-essential expression of *yisP*, the only phosphatase that catalyzes the conversion of FPP to farnesol, would also lead to less FPP consumption in this competing pathway, and the resulting extra FPP could be used by CrtMN enzymes to increase C_30_ carotenoid yield. In the Δ*yisP* mutant, known to exhibit no FPP phosphatase activity (Feng et al. 2014), excess FPP was distributed to increase the carotenoid yield in the engineered strain 1.39-fold (Fig. 2; Table 2). Thus, for the first time, the role of *yisP* knockout in an increased accumulation of C_30_ carotenoids in *B. subtilis* was demonstrated.

Cell engineering techniques have been previously used to improve C_30_ carotenoid productivity in *E. coli* and *B. subtilis*. *E. coli* strains were engineered to accumulate C_30_ carotenoids, with production levels ranging from 0.5 mg/ gDCW to 10.8 mg/L (Chae et al. 2010; Kim et al. 2010, 2022; Takemura et al. 2021). *B. subtilis* has also been engineered using two-plasmid systems comprising pHY_crtMN (Yoshida et al. 2009), mediating *crtMN* gene overexpression, and xylose-inducible pHCMC04G (Xue et al. 2015), mediating stable overexpression of all MEP pathway enzymes. In total, the yield of C_30_ carotenoids achieved was 21 mg/g DCW, the highest production in *B. subtilis* reported to date (Abdallah et al. 2020). In the present study, the combination of chromosomal overexpression of farnesyl diphosphate synthase, dehydrosqualene synthase and dehydrosqualene desaturase encoded by *fpps*, *crtM* and *crtN*, respectively, with the simultaneous disruption of the *yisP* gene, resulted in a titer of 9.11 mg/L C_30_ carotenoids, and a yield of 4.42 mg/g DCW. Although the C_30_ carotenoid accumulation is similar to that achieved in *E. coli* strains and lower (4.7-fold) than in *B. subtilis* overexpressing the eight enzymes of the MEP pathway, it should be noted that we only focused on improving the last three steps downstream of the MEP pathway. Consequently, one could expect that combining both strategies would serve to obtain a superior productive strain. Additionally, we demonstrated that routinely used industrial bacterial feed (antibiotic- and xylose-inducer-free) may provide a cost-effective bioprocess for the industrial production of C_30_ carotenoids. In a nutshell, taking advantage of its inherent capacity to synthesize C_30_ carotenoids, we have developed a plasmid-less, marker-free, *B. subtilis* strain that can serve as a stepping stone for further genetic engineering and fermentation process optimization targeted at a sustainable and efficient production of C_30_ carotenoids.

## Electronic supplementary material

Below is the link to the electronic supplementary material.

Supplementary Material 1

## Data Availability

The datasets generated during and/or analyzed during the current study are available from the corresponding author on reasonable request.
