# Supplementary material for: Metabolic engineering of Bacillus subtilis toward the efficient and stable production of C30-carotenoids
Source: AMB Express. 2023 Apr 29;13:38. doi: 10.1186/s13568-023-01542-x (PMC10148934; doi:10.1186/s13568-023-01542-x)
Supplement: Supplementary file 1 — Supplementary Material 1 [file 13568_2023_1542_MOESM1_ESM.pdf]

Metabolic engineering of *Bacillus subtilis* toward the efficient and stable production of C_{30}-carotenoids

Oriana Filluelo, Jordi Ferrando and Pere Picart

SUPPLEMENTARY MATERIAL

Figure S1: HPLC chromatogram and absorption spectra of C_{30} carotenoids

Table S1: Primers designed in this study

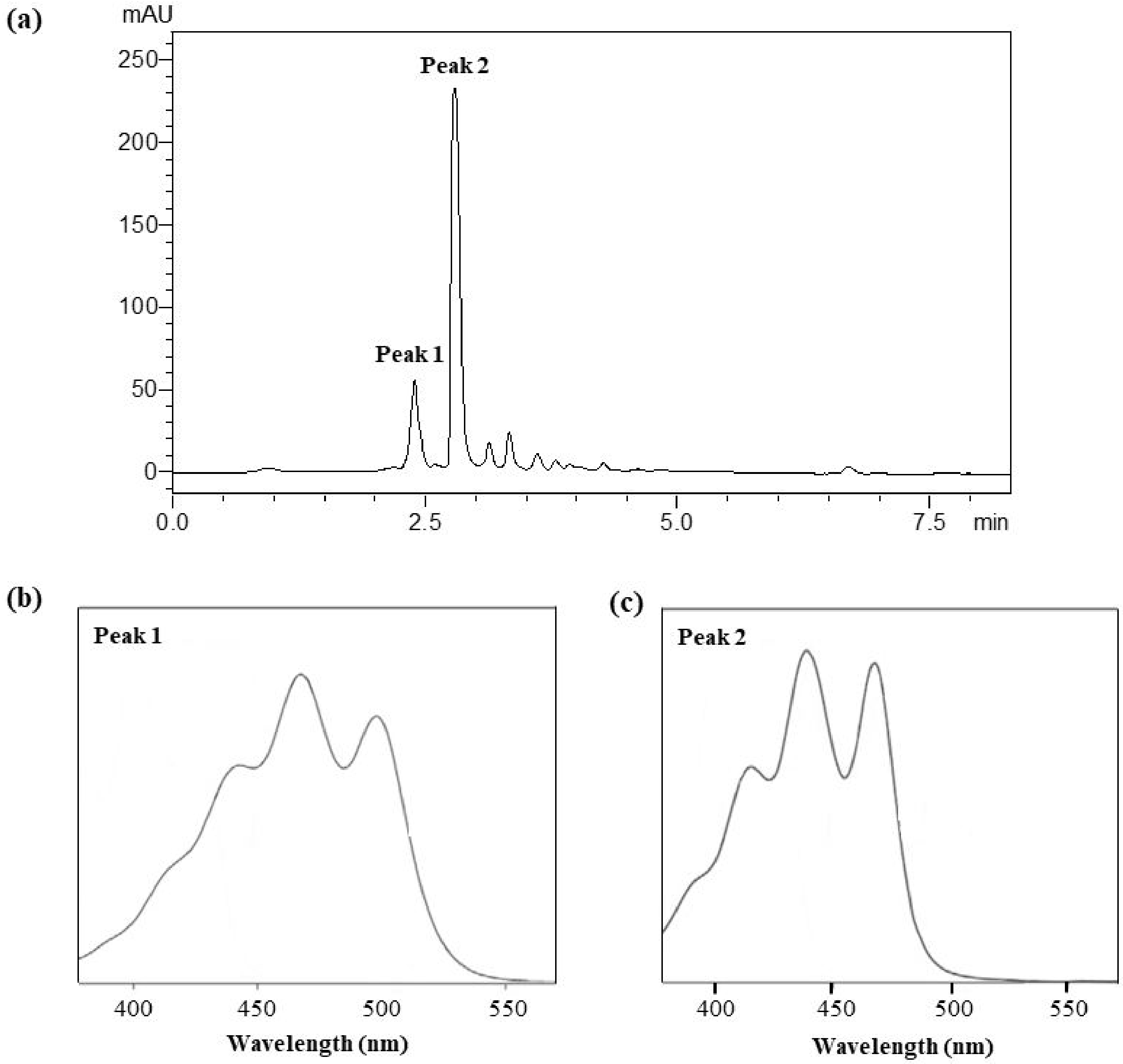

Figure S1. Analysis of C_{30} carotenoids produced by engineered *B. subtilis* strains. a) Chromatogram of C_{30} carotenoids separated by HPLC. The quantity of each C_{30} carotenoid was determined by comparing HPLC profiles peak areas to that of a β-carotene standard curve. The two major peaks correspond to 4,4'-diapolycopene (peak 1) and 4,4'-diaponeurosporene (peak 2) at 450 nm. b) UV/Vis absorption spectra for compound corresponding to individual peak 1 (4,4'-diapolycopene) and c) UV/Vis absorption spectra for compound corresponding to individual peak 2 (4,4'-diaponeurosporene).

Table S1. Primers designed in this study*.

Name	Sequence	Purpose
		**PCR for *crtMN* genes cloning into pBS0E**
**P1F**	5' – CCGGAATTCTAAGGAGGAAAAACATATGACAATGATGGATATGAATTT	PCR product containing *crtMN* genes flanked by *EcoRI* and *SpeI*
**P1R**	5' – GGACTAGTTTATACGCCCCGCTCAAT	
**TS1F**	5' – TACGTACATTTGACTGGGATACAC	**Target sequence for *sigX* gene replacement**
**TS1R**	5' – AAACGTGTATCCCAGTCAAATGTA	
		**PCR of homology template for *sigX* replacement for *fpps***
**P2F**	5' – AA**GGCCAACGAGGCC**AGTCATGAGCTGAGAACAC	upstream *sigX* gene homologous arm
**P2R**	5' – CATTTGAAACCCCTCCGTT	
**P3F**	5' – AACGGAGGGGTTTCAAATGGTGAATGAAATGAATTTAAAAGAG	*fpps* gene amplification
**P3R**	5' – TGAGGCGAACGATGGTCTTTAACTATCACGTTTTGCTATATA	
**P4F**	5' – AGACCATCGTTCGCCTCA	downstream *sigX* gene homologous arm
**P4R**	5' – AA**GGCCTTATTGGCC**AATCATCAACTTCTGACTCC	
**TS2F**	5' – TACGCGGTAAACAACATCTATGGA	**Target sequence for *YisP* gene deletion**
**TS2R**	5' – AAACTCCATAGATGTTGTTTACCG	
		**PCR of homology template for *YisP gene* deletion**
**P5F**	5' – AA**GGCCAACGAGGCC**AGAAGGATAGATGAGCAGGG	upstream *yisP* gene homologous arm
**P5R**	5' – CTCATTGCTGGTAAGCTTCTTT	
**P6F**	5' – AAAGAAGCTTACCAGCAATGAGAAAACACCCGGCCTTGATTGA	downstream *yisP* gene homologous arm
**P6R**	5' – AA**GGCCTTATTGGCC**TGGCCATGTCCACATCTATC	

* *Sfi*I restriction sites are indicated in **bold**. *Eco*RI restriction site is underlined and S*pe*I restriction site is doble underlined
